# Improvement of Bearing Capacity in Recycled Aggregates Suitable for Use as Unbound Road Sub-Base

**DOI:** 10.3390/ma8125493

**Published:** 2015-12-16

**Authors:** Laura Garach, Mónica López, Francisco Agrela, Javier Ordóñez, Javier Alegre, José Antonio Moya

**Affiliations:** 1 Department of Civil Engineering, University of Granada, Severo Ochoa s/n, Granada 18071, Spain; 2 Department of Construction and Project Engineering, University of Granada, Severo Ochoa s/n, Granada 18071, Spain; mlopeza@ugr.es (M.L.); javiord@ugr.es (J.O.); fjalegre@ugr.es (J.A.); 3 Area of Construction Engineering, University of Córdoba, Ed. Leonardo Da Vinci, Campus Rabanales, Ctra N-IV, Km-396, Córdoba 14014, Spain; fagrela@uco.es; 4 Ministry of Development, Avda Madrid, 7, Granada 18071, Spain; jamoya@fomento.es

**Keywords:** self-cementing, mechanical properties, recycled aggregates, pozzolanic reactions, electronic microscopy

## Abstract

Recycled concrete aggregates and mixed recycled aggregates are specified as types of aggregates with lower densities, higher water absorption capacities, and lower mechanical strength than natural aggregates. In this paper, the mechanical behaviour and microstructural properties of natural aggregates, recycled concrete aggregates and mixed recycled aggregates were compared. Different specimens of unbound recycled mixtures demonstrated increased resistance properties. The formation of new cement hydrated particles was observed, and pozzolanic reactions were discovered by electronon microscopy in these novel materials. The properties of recycled concrete aggregates and mixed recycled aggregates suggest that these recycled materials can be used in unbound road layers to improve their mechanical behaviour in the long term.

## 1. Introduction

In recent years, the use of construction and demolition waste (CDW) in civil engineering has increased considerably, and recycled aggregates (RA) are widely used as subbase materials in production [1].

Two types of RA can be generated from CDW: recycled concrete aggregates (RCA), which contain particles primarily originating from recycled crushed concrete [2], and mixed recycled aggregates (MRA), which are produced from recycled crushed masonry and include brick, mortar, concrete, asphalt and gypsum particles [3].

Several studies have concluded that RCA exhibit lower density and higher water absorption than natural aggregates, and if this material is applied in concrete, its mechanical behaviour is reduced [4,5,6]. However, the mechanical behaviour of MRA is appropriated in unbound road layers and cement-treated road layers [7,8,9].

Mixed recycled aggregates have been studied, especially in non-structural concrete applications [10,11]. The densities of these recycled materials are lower than that of RCA, and MRA’s water absorption is higher than that of RCA. Because it can affect the mechanical behaviour of concrete mixtures or cement-treated materials produced using MRA, the sulphate content of these materials must be controlled [12,13].

The use of RCA and MRA in pavement layers has been analysed in several studies. Molenaar and Nierkerk [14] studied the behaviour (e.g., gradation, composition, *etc.*) of unbound base course materials generated from recycled concrete and masonry rubble. The results demonstrated that, due to the good quality of these materials, they could be applied in road bases. Poon and Chan [15] studied the possibility of using RCA and crushed clay brick as aggregates in unbound subbase materials, and the results indicated that these recycled materials (MRA) could feasibly be blended to build road subbases. Azam and Cameron [16] studied the optimal application of a blend with 20% MRA and 80% RCA, and this mixture exhibited satisfactory properties for application in the construction of unbound granular pavements.

In real-world applications, Herrador, *et al.* [17] analysed the behaviour of RCA in a section of a road under real vehicle traffic conditions. Jiménez, *et al.* [18] evaluated different types of recycled aggregates from CDW as granular materials for the construction of unbound rural path sub-bases. In both cases, the behaviour of these materials was excellent and improved in the long term. MRA were studied, and high sulphur contents and poor fragmentation resistances were observed.

Agrela, *et al.* [19] applied MRA obtained from a mixture of concrete and masonry work in the study of cement-treated granular materials. All (100%) of the natural material was substituted by the selected MRA, and the structural layers of the road demonstrated appropriate bearing capacities. Additionally, the road surface did not exhibit any deformations two years after its construction. Perez, *et al.* [9] studied the efficiency of using RCA treated with cement in the construction of a road in Málaga (Spain).

In general, the studies of MRA and RCA application in road bases and sub-bases have concluded that the long-term effectiveness of the road sections built using recycled aggregates was similar to that of sections constructed using natural aggregates. Some authors have argued that the observed increase in the strength of the sub-base materials prepared with RCA is caused by the self-cementing phenomenon. Arm [20] studied the expected growth in stiffness, which is a result of self-cementing properties, in layers of crushed concrete from demolished structures. Poon, *et al.* [21] suggested that the anhydrated cement remaining in the mortar of fine recycled concrete aggregates (<5 mm) was the principal cause of the self-cementing process. Other authors demonstrated that the combined presence of concrete and ceramic materials induces pozzolanic reactions, which contribute to an increase in the bearing capacity of the compacted mixed recycled aggregate [22]. Likewise, studies carried out by Khatib [23] on the incorporation of ceramic fines into concrete showed that they provided greater relative resistance to compression after 90 days of curing as a consequence of the maximum effect of the pozzolanic reaction between the silica and alumina of the ceramic fines and the hydrated portlandite of the cement.

In this study, three materials were analysed and compared in the laboratory: recycled concrete aggregates (RCA), mixed recycled aggregates (MRA) and materials prepared with natural aggregates (NA). The evolutions of their mechanical characteristics were studied over time using the California Bearing Ratio (CBR) test and the accelerated swelling test. Diffraction and microscopy tests were applied to determine the underlying causes of the improvement in compressive strength or the self-cementing process observed in recycled aggregates.

## 2. Materials

Three types of aggregates were used in this research: natural aggregates (NA), recycled concrete aggregates (RCA) and mixed recycled aggregates (MRA). The predominant materials in the RCA were unbound aggregates, concrete and mortar and asphalt. The MRA contains the same materials as RCA and ceramics.

Several tests were conducted to analyse the different materials. These tests included evaluations of the particle size distribution, water absorption, Angeles abrasion value, Atterberg limits and flakiness index.

### 2.1. Natural Aggregates (NA)

In this study, the natural aggregate used was composed of dolomite. Its properties are summarised in Table 1.

**Table 1:** Properties of the natural and recycled aggregates.

Properties	NA	RCA	MRA	Standard Established Limits
Density SSD (Mmg/m^3^) (0.063–4) (UNE-EN 1097-6:2001)	2.77	2.68	2.54	–
Density SSD (Mmg/m^3^) (4–31.5) (UNE-EN 1097-6:2001)	2.76	2.63	2.47	–
Water absorption (%) (0.063–4) (UNE-EN 1097-6:2001)	1.96	2.45	5.83	–
Water absorption (%) (4–31.5) (UNE-EN 1097-6:2001)	0.54	2.37	5.01	–
Nominal size (mm)	25	25	25	–
Sand Equivalent (UNE EN 933-8:2000)	38	52	46	T3 to T4: SE > 35
L.A. abrasion value (%) (UNE EN 1097-2:1999)	24.00	32.92	36.40	T3 to T4: <35% *
Flakiness index (UNE EN 933-3:1997)	13	5	13	<35%
Acid-soluble sulphate (%SO_3_) (UNE 1744-1:1999)	0.05	0.53	0.70	0.5%(c); <1%(d)
Organic matter (%) (UNE 103204:1993)	0	0.75	0.43	<1%

T3 = [50–200) heavy vehicles/day; T4 = [0–50) heavy vehicles/day; Medium Intensity—Daily (vehicles/day); * 40% in recycled aggregates; c: Materials in contact with cement-treated layers; d: Remaining cases.

**Figure 1:**
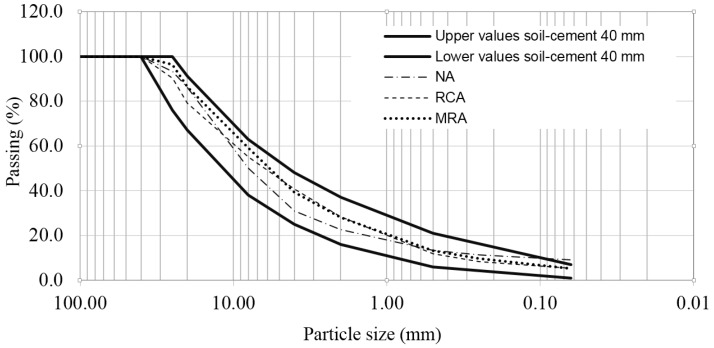
Particle size distribution curves of NA, RCA and MRA, compared with the granulometric limits.

The particle size distributions were analysed in accordance with the Spanish standard UNE-EN-933-1 [24]. Figure 1 shows the particle size distribution curves of the NA, RCA and MRA. Additionally, the grading curve range of the material termed “soil-cement 40 mm” is represented. This material is termed SC-40 by the Spanish General Technical Specifications for Road Construction [25].

### 2.2. Recycled Aggregates (RA): Recycled Concrete Aggregates (RCA) and Mixed Recycled Aggregates (MRA)

The recycled aggregates were obtained from a recycling plant (Inerts Aggregate Guhilar) situated in Granada, Spain. The properties of the RCA and MRA are summarized in Table 1. Analyzing the properties (Table 1) and the particle size distributions (Figure 1), the results were obtained according to UNE-EN-933-11 [26].

The NA, RCA and MRA were well-graded. The particle size distributions of the three materials were between the low and high gradient limits, indicating that, in general, the NA, RCA and MRA did not contain excess amounts of coarse or fine particles. The RCA and MRA were close to the lower gradient limit for particle sizes between 20 and 25, and the NA was close to the higher gradient limit for these particle sizes (25).

The size distribution curves of the materials were continuous, indicating more opportunities for interactions between particles and the ability to obtain a greater degree of compaction. The fine fraction content (<0.063 mm) was less than 10% for the RCA, MRA, and NA, although the percentage in NA was closer to 10%.

The three materials exhibited similar saturated surface-dry density (SSD) values for the 0.063–4 mm fractions (Table 1). The NA showed a slightly higher density value, followed by the RCA and MRA for the 4–31.5 mm fraction. NA exhibited reduced water absorption, compared with the two recycled aggregates [27].

The differences between the RCA and MRA were primarily due to mortar adhesion and to the presence of ceramic particles (masonry). The RCA had the Angeles coefficient lower than 35% (32.92%) fulfilling the limit established by Spanish regulation [25]. The MRA obtained value (36.40%) was also lower than the limit value established by Spanish regulation (40%) for recycled aggregates. Other authors obtained values of around 40% [18,20]. Los Angeles coefficient is identified as one of the most critical properties of recycled aggregates from mixed debris. The NA had the lowest Angeles coefficient (24%). The RCA exhibited a lower flakiness index value than the NA and MRA index because it contains more rounded particles.

The NA displayed the lowest sand equivalent (SE) value. The RCA contained the most organic impurities, and the NA contained the least. Spanish regulations [25] specify a maximum amount of acid-soluble sulphates that can be present in aggregates (limit value = 0.8%). The soluble sulphate content, which is partly caused by the mortar-adhered particles, can lead to swelling problems associated with the formation of ettringite [12,13,14,15,16,17,18,19,20,21,22,23,24,25,26,27,28]. Therefore, it is important to control the sulphate content to avoid exceeding the admissible limits by selecting the residue at the source and using an appropriate treatment in the processing plant. In the NA, this content was practically zero. In contrast, in the MRA, the acid-soluble sulphate content was close to 0.8% by weight, which is the limit prescribed by the Spanish regulations and other national and international rules [25,29].

The results indicate that the incorporation of masonry particles increased the acid-soluble sulphate content in the MRA to a limited extent. The sulphate content must be controlled to avoid exceeding the admissible limits and may be altered by selecting the residue at the source and using an appropriate treatment in the processing plant.

RCA and MRA may contain more gypsum particles, resulting in an increased soluble sulphate content, which is usually a limiting factor in the use of NA [30]. The soluble sulphate content is strongly influenced by the gypsum percentage fixed to the masonry particles in the MRA [3]. The sulphate content was less than 0.8% in the evaluated samples because the recycled materials were subjected to a selection process that included the removal of a large amount of impurities.

The percentages by weight of the residues that each type of RA comprises are listed in Table 2. The aggregates were composed primarily of asphalt, ceramics, concrete and mortar, natural aggregates and impurities such as wood, glass, plastic and metal.

**Table 2:** Composition of recycled aggregates evaluated according to UNE-EN-933-11.

Properties	RCA	MRA
Ra (%) (Asphalt)	8.9	8.1
Rb (%) (Ceramics)	0.3	23.7
Rc (%) (Concrete and mortar ^a^)	42.1	31.5
Ru (Unbound aggregates ^b^)	48.5	36.5
X_1_ (%) (Natural soil)	0	0
X_2_ (%) (Others)	<0.1	<0.1
X_3_ (%) (Gypsum)	<0.1	<0.1

^a^ Natural aggregates with cement mortar attached; ^b^ Natural aggregates without cement mortar attached.

The RCA was considered to be very pure because more than 90% of the content consisted of natural aggregates with and without mortar from concrete waste. The predominant materials in the MRA were unbound aggregates, concrete and ceramics. The ceramic contents were <10% in the RCA and <30% in the MRA [27]. Both recycled aggregates contained less than 1% impurities. The high asphalt content in both materials, RCA and MRA, came from the plant where they were manufactured, which incorporates large amounts of asphalt into the manufactured mixtures.

## 3. Experimental Tests

The following experimental tests were applied to study the short- and long-term mechanical behaviours and to evaluate the self-cementing capacity of the recycled concrete aggregates and mixed recycled aggregates.

### 3.1. Modified Proctor Test

An analysis of the compaction characteristics of the recycled aggregates with varying compaction moistures was performed according to the UNE-EN 103501 [31] modified Proctor test. The test was performed using the modified Proctor mould, which consisted of a cylinder 152.5 mm in diameter and 129.8 mm in length. All of the materials were compacted in five layers, with 60 blows applied to every layer.

The moisture-density curves, representing the changes between moisture content and the dry density in the material, were obtained. The percentage of moisture content corresponding to the maximum dry density on the moisture content/dry density curve is the optimum value.

### 3.2. California Bearing Ratio (CBR) Standard Test

The California Bearing Ratio (CBR) is defined as the ratio of the force required to penetrate a circular piston of 1935 mm^2^ cross section into soil in the CBR mold at a rate of about 1 mm/min, to that required for similar penetration into a standard sample of compacted crushed rock—13.24 kN and 19.96 kN at penetrations of 2.50 mm and 5.00 mm, respectively. This ratio is determined at these two penetrations as follows: CBR = measured force/standard force × 100. The higher of these two values is reported as the CBR value for that specimen [32].

The CBR test provides an indirect measurement of the shear strength and is extremely dependent on the moisture content and on the level of compaction.

The soaked CBR tests were carried out on specimens subjected to a modified Proctor compaction process at the optimum water content. The three materials were soaked for 4, 28, 60 or 180 days, and then they were compacted at their corresponding optimum moisture contents.

### 3.3. Accelerated Swelling Test for Soil Treated by Lime and/or Hydraulic Binder According to UNE-EN-13286-49

An accelerated swelling test was performed on the three aggregates (NA, RCA and MRA) according to UNE-EN-13286-49 [33]. This test consisted of the preparation of three compacted specimens in a cylindrical mould with diameter and length of 5 mm. After the compaction process, the specimens were cured at 20 °C and a relative humidity of 95% for three days. Then, the specimens were submerged in 40 °C water for 7 days. After this week, the swelling and compressive strengths of the samples were measured in the laboratory.

### 3.4. X-ray Diffraction (XRD) and Scanning Electron Microscopy (SEM)

The scanning electron microscopy (SEM) analysis was completed using a Bruker D8 Advance. The analysis was used for solid material characterisation and was performed by using a scanning electron microscope. The signals generated during the analysis produced two-dimensional images, which revealed information about the tested samples, including their external morphologies (textures). The present study also estimated the composition of the samples using X-ray spectroscopy.

These tests were applied to the NA, RCA and MRA and to several processed samples from these materials.

## 4. Results and Discussion

### 4.1. Modified Proctor Test

**Figure 2:**
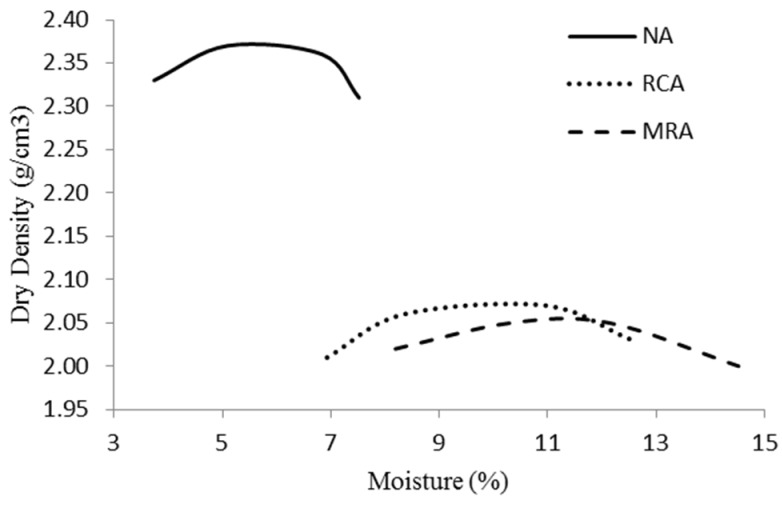
Modified Proctor curves for the three materials.

Figure 2 shows the moisture-density curves. Table 3 shows the dry density values and the optimum moisture content obtained with the modified proctor test for the three materials. According to Poon, *et al.* [21], the difference in the maximum dry density and the optimum moisture content in recycled aggregates is mainly caused by the density and the high water absorption capacity of materials. The moisture-density curves are an indication of the sensitivity of the density regarding the variations of moisture content for the materials. Materials with flat curves can tolerate a greater amount of variation in the moisture content without compromising much of the achieved density from compaction. Conversely, materials with sharp curves are very sensitive to the change in the moisture content and there is a need to ensure that the moisture content is close to the optimum value during compaction [15,18]. Figure 2 shows that all the materials exhibited curves that were very sensitive to changes in the moisture content, demonstrating the necessity of ensuring that the moisture content is close to the optimum value during compaction. NA showed a sharp curve where RCA and MRA showed flat curves.

**Table 3:** Optimum moisture content and maximum dry density: Modified Proctor test.

Blend	Optimum Moisture Content (%)	Maximum Dry Density (g/cm^3^)
NA	5.96	2.38
RCA	10.18	2.08
MRA	11.20	2.02

The densities obtained from the modified Proctor test were lower for the RCA and MRA than for the NA in agreement with other studies [15,19]. The optimum compaction moisture content was higher in the recycled materials than in the natural material. The results obtained show trends that are similar to those obtained in the work carried out by several authors [5,6,34]. Therefore, the NA had the highest maximum dry density and the lowest optimum moisture content. The difference in the maximum dry density and the optimum moisture content was mainly attributed to the physical properties of NA which had the highest particle density and was less porous compared to recycled aggregate [15]. On the other hand, the incorporation of brick (MRA) increased the optimum moisture content and decreased the maximum dry density (Figure 2 and Table 3) as a result of the high water absorption and the low particle density of the crushed brick particles. The difference between RCA and MRA was mainly caused by the difference in the density and the water absorption between these two materials.

### 4.2. California Bearing Ratio (CBR) Standard Test

The CBR tests were carried out under different conditions: in unsoaked and in 4-day, 28-day, 60-day and 180-day soaked conditions.

**Figure 3:**
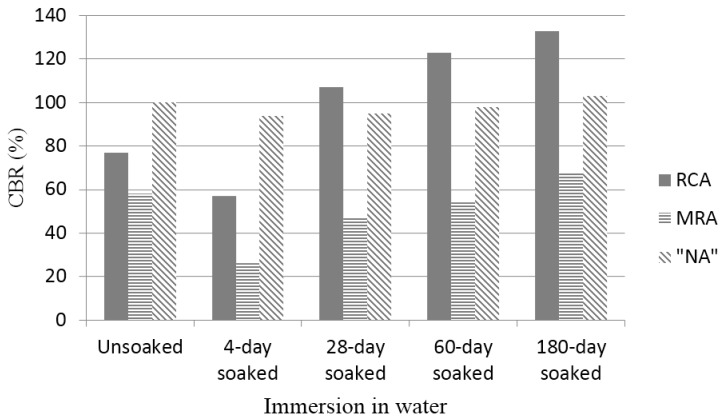
CBR values (unsoaked, 4-day soaked, 28-day soaked, 60-day soaked and 180-day soaked).

The results are summarised in Figure 3.

As shown in Figure 3, the CBR values of NA, RCA and MRA decrease when the materials are immersed in water [15]. In unsoaked and in 4-day soaking conditions, the NA composed of natural materials exhibited the highest CBR values. A lower CBR values was obtained for the MRA in 4-day soaking conditions. One possible reason for this difference is the lower intrinsic particle strength of crushed clay brick, which could have resulted in a decrease in the overall bearing strength of the materials.

**Figure 4:**
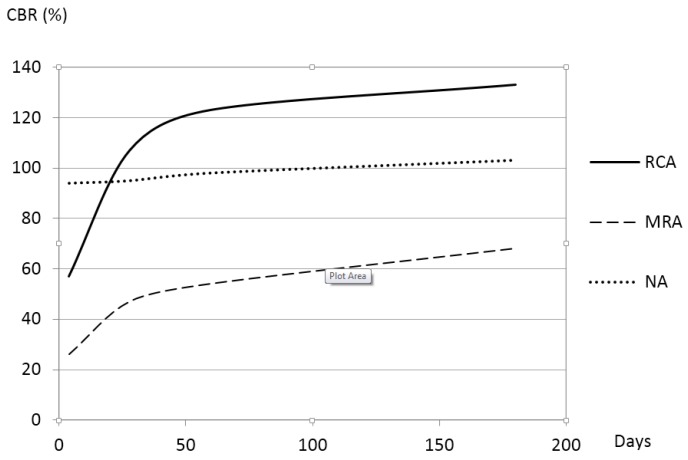
CBR evolution in soaked conditions.

Figure 4 shows the CBR values of the three materials in the soaked conditions. The minimum CBR necessary for the use of these recycled materials in unbound road layers is 20% in Spain [25].

The CBR values of all of the materials increased with time. At the beginning of the study, the CBR value of the NA was high and remained practically constant over time. The CBR value of the RCA was lower than that of the NA but reached a similar value after approximately 28 days. Under 180-day soaking conditions, the CBR value of the RCA increased to a maximum of 133. The NA and RCA exhibited in soaked CBR values higher than the cut-off value in Spain (20%). Even these values were higher than 30%, considered as an appropriate value of CBR to apply in unbound materials in road layers in other countries [15]. However, the MRA sample reached this value after approximately seven days.

As shown in Figure 3 and Figure 4, the RCA and MRA demonstrated important increases in their CBR values at 28 days. In fact, the RCA reached 80% of its compressive strength at 28 days (from a value of 107 at 28 days to a value of 133 at 180 days) and MRA reached 69.12% of its compressive strength at 28 days (from a value of 47 at 28 days to a value of 68 at 180 days). These significant increases in the compressive strength of the RCA and MRA at 28 days are likely due to the self-cementing of the anhydrated cement remaining in the recycled concrete aggregates, as several authors indicated in their research. According to Poon and Chan [15] and Shui, *et al* [35], the remaining cement in the adhered mortar of the fine recycled concrete aggregates is the main cause of the self-cementing effect noticed on the sub-bases made with recycled aggregates. According to Vegas, *et al.* [22], the increase in the bearing capacity in MRA may be attributed to either the pozzolanic reactions or to the remaining cement contained in the recycled concrete aggregates.

In Figure 4, it can be observed that the long-term behaviour of the RCA is superior to that of the NA. MRA exhibit lower mechanical strength than NA and RCA, according to Katz [4]. Although the CBR value of the MRA was lower than that of the RCA or NA, the extent of the disparity between the RCA and NA decreased with time.

Furthermore, all of the materials demonstrated negligible swelling (*i.e*., <0.05%) after a 4-day soaking period and should not cause any cohesion problems during saturation conditions. In Spain, CBR values >20% are considered suitable for use as a sub-base; in Hong Kong, these values should be >30%. The results indicated that 100% RCA or MRA is adequate for use as a granular sub-base material.

### 4.3. Accelerated Swelling Test

Six samples of each material were prepared, and all of the samples were compacted in a mould (d = h = 50 mm) according to UNE-EN-13286-49 [33]. As indicated in the methodology, the compacted samples were cured in a moist chamber at 20 °C for three days and then were immersed in water for seven days at 40 °C. Table 4 shows the results of the accelerated swelling test.

**Table 4:** Accelerated swelling test for NA, RCA and MRA.

Blends	Initial Density (g/m^3^)	Compressive Strength (MPa)	Swelling after 7 Days of Soaking (%)
NA	2.37	0.04	2.60
RCA	2.16	0.55	2.30
MRA	2.14	1.15	4.00

As shown in Table 4, the MRA showed the best compressive strength value. It was two-fold greater than the RCA’s compression strength value, which is twice that of the highest compressive strengths obtained for the NA. According to Salem and Burdette [36] and Etxeberria, *et al.* [37], the observed increases in the compressive strength of recycled aggregates is due to the rough texture and absorption capacity of the adhered mortar in these materials that provides better bonding and interlocking between the cement paste and the recycled aggregates themselves. The high compressive strengths obtained for the RCA and MRA may be caused by the self-cementing process that occurred in the two materials. This self-cementing process may be due to the anhydrated cement remaining or to the agglomeration from chemical reactions (such as pozzolanic reactions) found within these materials. According to Vega, *et al.* [22], the generation of greater amounts of ceramic fines has a favourable effect on the process of the consolidation of the granular material because, in the presence of water, fine particles of a ceramic nature induce pozzolanic reactions with the calcium hydroxide in the concrete. This possibility was analysed by SEM and X-ray diffraction (see below). Despite the phenomenal increase in the compressive strength of the MRA, the very high degree of swelling (4%) exhibited by this material can invalidate its use as a road construction material. In order for this material to be valid, its swelling would have to be reduced, and therefore its percentage of ceramic material content would have to be limited (likely to be approximately 15%). According to the results observed in Table 4, the material that demonstrated the best behaviour, because it showed the best values considering the combination of compressive strength and swelling, was the RCA, followed by the NA. The worst material was the MRA due to its high degree of swelling. RCA also exhibited the best results in the CBR test.

Another remarkable factor is the importance of the pre-wetting and compaction in achieving the highest possible efficiency of these graded aggregates [18,22]. The compressive strengths of the samples were reduced under different moisture and compaction conditions (unsoaked and compacted 50% in the modified Proctor test).

### 4.4. SEM and Diffraction Analyses of the Blended Mixtures

SEM and diffraction analyses were performed to explain the considerable increase in the compression strength exhibited by the MRA. This increase may have resulted from the agglomeration process generated by chemical reactions within the MRA, in addition to the self-cementing process that likely occurred in the recycled aggregates.

**Figure 5:**
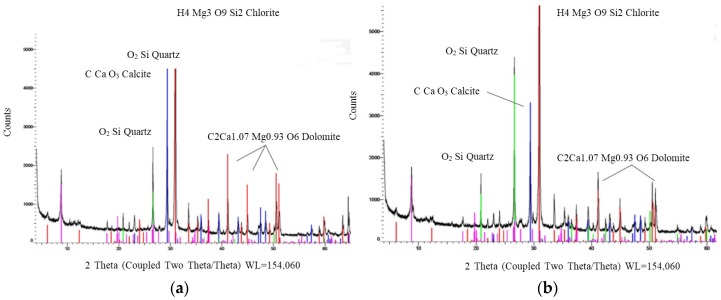
(**a**) X-ray diffraction patterns of RCA; and (**b**) MRA.

The X-ray diffraction patterns of the RCA and MRA are shown in Figure 5a,b, respectively.

The results indicated that both samples contained similar components. In particular, the quantity of calcite appears to be higher in the RCA than in the MRA because the former contains more cement. The main difference between the two spectra is the increase in quartz in the MRA sample compared to the RCA sample. This increase is obviously due to the incorporation of brick in the sample. Diffraction showed a slight difference in the compositions of the two materials. However, diffraction did not show if new unions among components in MRA had occurred. These bonds could produce an increase in the MRA compressive strength, with respect to the RCA compressive strength. Thus, electron microscopy tests were performed.

New small crystals were found in the microscopic analysis which may have formed from the anhydrated cement remaining in both materials (RCA and MRA). The compaction of the materials would aid in the self-cementing process.

**Figure 6:**
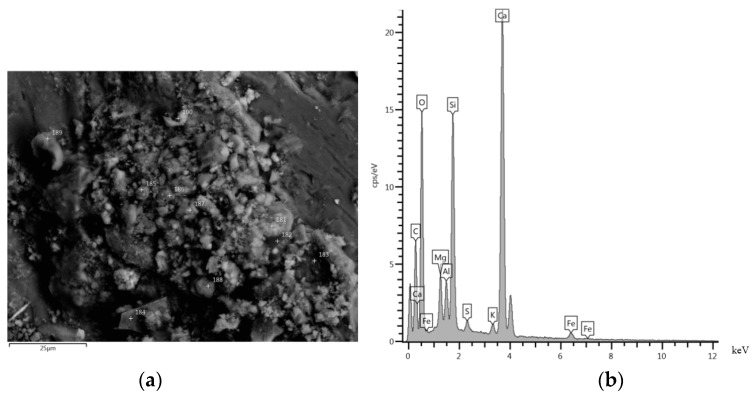
(**a**) SEM micrograph image of RCA; and (**b**) composition of an RCA sample estimated by X-ray spectroscopy.

**Figure 7:**
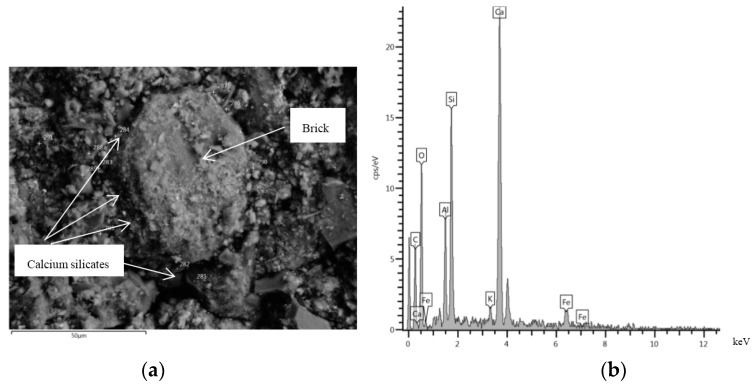
(**a**) SEM micrograph image of MRA; and (**b**) composition of an MRA sample estimated by X-ray spectroscopy.

Figure 6 and Figure 7 show the SEM images and the compositions estimated by X-ray spectroscopy for the RCA and MRA, respectively.

Component composition was analysed in both materials by microscopy. Calcium silicates were found in both samples. It is likely that the remaining anhydrated cement in both recycled concrete aggregates was the principal cause of the RCA and MRA with greater strength [4,36,37,38]. In addition, microscopy revealed the formation of new crystals in the MRA. A large proportion of calcium silicates was found around the brick (Figure 7a).

According to Vegas, *et al.* [22], the fine particles of a ceramic nature induce pozzolanic reactions with the calcium hydroxide in the concrete. Therefore, the presence of concrete and ceramic materials in the MRA probably induced pozzolanic reactions that increased the material’s resistance to simple compressive strength, providing the MRA with twice the strength of the RCA. This fact should be further investigated in future research.

## 5. Conclusions

In this research, the mechanical behaviour of three materials—a recycled concrete aggregate (RCA), a mixed recycled aggregate (MRA) and a natural aggregate (NA)—were analysed. The results demonstrated that the RCA and MRA exhibit lower densities and higher water absorption than the NA.

The NA reached a high load capacity value at the beginning of the analysis, which continued to increase over time. However, the RCA and MRA demonstrated improved load capacity over time. The main improvement for both recycled materials occurred at 28 days. This result appears to be associated with the presence of anhydrated cement in both samples.

The increase in the load capacity for RCA was higher due to the high quantity of cement in the sample. The RCA had the highest load capacity at 180 days; and the MRA had the lowest load capacity.

The accelerated swelling test, in which the samples were submerged in 40 °C water for seven days, indicated that the MRA displayed the best compressive strength value. This value was double the compression strength value of the RCA, which, in turn, was twice that of the highest compressive strengths obtained for the NA. Despite the surprising increase in the compressive strength of the MRA, the swelling value (4%) observed for the material, which likely resulted from its high aluminium content, could invalidate the use of this material in road construction. Thus, the RCA was the material that demonstrated the best results in the accelerated swelling test and exhibited both high compressive strengths and a low degree of swelling.

According to the results obtained using electronic microscopy, the increase in the compressive strength of the MRA may have been due to the pozzolanic reactions generated by the presence of concrete and ceramic materials.

In general, the RCA demonstrated the best long-term behaviour due to its particle resistance, increase in load capacity, high compressive strength and low swelling. The MRA exhibited high porosity and anhydrated old cement content and displayed increased medium-term load capacity.

## References

[1] Leite F.D.C., Motta R.D.S., Vasconcelos K.L., Bernucci L. (2011). Laboratory evaluation of recycled construction and demolition waste for pavements. Constr. Build. Mater..

[2] Kou S.C., Poon C.S. (2009). Properties of self-compacting concrete prepared with recycled glass aggregate. Cem. Concr. Compos..

[3] Barbudo A., Agrela F., Ayuso J., Jiménez J.R., Poon C.S. (2012). Statistical analysis of recycled aggregates derived from different sources for sub-base applications. Constr. Build. Mater..

[4] Katz A. (2003). Properties of concrete made with recycled aggregate from partially hydrated old concrete. Cem. Concr. Res..

[5] Tabsh S.W., Abdelfatah A.S. (2009). Influence of recycled aggregate on strength properties of Concrete. Constr. Build. Mater..

[6] Rao M.C., Bhattacharyya S.K., Barai S.V. (2011). Influence of field recycled coarse aggregate on properties of concrete. Mater. Struct..

[7] Jiménez J.R., Ayuso J., Galvín A.P., López M., Agrela F. (2012). Use of mixed recycled aggregates with a low embodied energy from non-selected CDW in unpaved rural roads. Constr. Build. Mater..

[8] Xuan D.X., Houben L.J.M., Molenaar A.A.A., Shui Z.H. (2012). Mechanical properties of cement-treated aggregate material—A review. Mater. Des..

[9] Perez P., Agrela F., Herrador R., Ordoñez J. (2013). Application of cement-treated recycled materials in the construction of a section of road in Malaga, Spain. Constr. Build. Mater..

[10] Brito J., Pereira A.S., Correia J.R. (2005). Mechanical behaviour of non-structural concrete made with recycled ceramic aggregates. Cem. Concr. Compos..

[11] Binici H. (2007). Effect of crushed ceramic and basaltic pumice as fine aggregates on concrete mortars properties. Constr. Build. Mater..

[12] Agrela F., Cabrera M., Galvín A.P., Barbudo A., Ramirez A. (2014). Influence of the sulphate content of recycled aggregates on the properties of cement-treated granular materials using Sulphate-Resistant Portland Cement. Constr. Build. Mater..

[13] Mas B., Cladera A., del Olmo T., Pitarch F. (2012). Influence of the amount of mixed recycled aggregates on the properties of concrete for non-structural use. Constr. Build. Mater..

[14] Molenaar A.A.A., van Niekerk A.A. (2002). Effects of gradation, composition, and degree of compaction on the mechanical characteristics of recycled unbound materials. Transp. Res. Record.

[15] Poon C.S., Chan D. (2006). Feasible use of recycled concrete aggregates and crushed clay brick as unbound road sub-base. Constr. Build. Mater..

[16] Azam A.M., Cameron D.A. (2013). Geotechnical properties of blends of recycled clay masonry and recycled concrete aggregates in unbound pavement construction. J. Mater. Civil Eng..

[17] Herrador R., Pérez P., Garach L., Ordóñez J. (2012). Use of recycled construction and demolition waste aggregate for road course surfacing. J. Transp. Eng..

[18] Jiménez J.R., Agrela F., Ayuso J., López M. (2011). A comparative study of recycled aggregates from concrete and mixed debris as material for unbound road sub-base. Mater. Constr..

[19] Agrela F., Barbudo A., Ramírez A., Ayuso J., Carvajal M.D., Jiménez J.R. (2012). Construction of road sections using mixed recycled aggregates treated with cement in Málaga, Spain. Resour. Conserv. Recycl..

[20] Arm M. (2001). Self-cementing properties of crushed demolished concrete in unbound layers: Results from triaxial tests and field tests. Waste Manag..

[21] Poon C.-S., Qiao X.C., Chan D. (2006). The cause and influence of self-cementing properties of fine recycled concrete aggregates on the properties of unbound sub-base. Waste Manag..

[22] Vegas I., Ibañez J.A., Lisbona A., Sáez de Cortazar A., Frías M. (2011). Pre-normative research on the use of mixed recycled aggregates in unbound road sections. Constr. Build. Mater..

[23] Khatib J.M. (2005). Properties of concrete incorporating fine recycled aggregate. Cem. Concr. Res..

[24] (2006). UNE-EN 933–1: 2006. Test for Geometrical Properties of Aggregates. Part 1. Determination of Particle Size Distribution. Sieving Method.

[25] (2004). Spanish General Technical Specifications for Road Construction (PG3), 2004.

[26] (2009). UNE-EN 933–11: 2009. Tests for Geometrical Properties of Aggregates. Part 11: Classification Test for the Constituents of Coarse Recycled Aggregate.

[27] Agrela F., Sánchez de Juan M., Ayuso J., Geraldes V.L., Jiménez J.R. (2011). Limiting properties in the characterisation of mixed recycled aggregates for use in the manufacture of concrete. Constr. Build. Mater..

[28] Odler I., Colan-Subauste J. (1999). Investigations on cement expansion associated with ettringite formation. Cem. Concr. Res..

[29] (2002). BS 8500–2:2002: Concrete-Complementary British Standard to BS EN 206–1. Part 2: Specification for Constituent Materials and Concrete.

[30] Vegas I., Ibañez J.A., San José J.T., Urzelai A. (2008). Construction demolition wastes, Waelzslag and MSWI bottom ash: A comparative technical analysis as material for road construction. Waste Manag..

[31] (1994). UNE 103501:1994. Geotechnic. Compactation Test. Modified Proctor.

[32] Al-Amoudi O.S.B., Asi I.M., Al-Abdul Wahhab H.I., Khan Z.A. (2002). Clegg hammer—California-bearing ratio correlations. J. Mater. Civil Eng..

[33] (2008). UNE-EN-13286–49:2008. Accelerated Swelling Test for Soil Treated by Lime or Hydraulic Binder.

[34] Güneyisi E., Gesoğlu M., Algin Z., Yazici H. (2014). Effect of surface treatment methods on the properties of self-compacting concrete with recycled aggregates. Constr. Build. Mater..

[35] Shui Z., Xuan D., Wan H., Cao B. (2008). Rehydration reactivity of recycled mortar from concrete waste experienced to thermal treatment. Constr. Build. Mater..

[36] Salem R.M., Burdette E.G. (1998). Role of chemical and mineral admixtures on physical properties and frost-resistance of recycled aggregate concrete. ACI Mater. J..

[37] Etxeberria M., Vázquez E., Marí A., Barra M. (2007). Influence of amount of recycled coarse aggregates and production process on properties of recycled aggregate concrete. Cem. Concr. Res..

[38] Kou S.-C., Poon C.-S., Etxeberria M. (2011). Influence of recycled aggregates on long term mechanical properties and pore size distribution of concrete. Cem. Concr. Compos..

